# Management options for accidental injection of epinephrine from an autoinjector: a case report

**DOI:** 10.4076/1752-1947-3-7268

**Published:** 2009-06-08

**Authors:** Christian Mathez, Bernard Favrat, Philippe Staeger

**Affiliations:** 1 Department of Ambulatory Care and Community Medicine, University of Lausanne, Bugnon 44, 1011 Lausanne-CHUV, Switzerland

## Abstract

**Introduction:**

Epinephrine autoinjector devices are used with increasing frequency to treat severe anaphylactic reactions. Accidental injection, usually involving a finger, is a potential complication.

**Case presentation:**

A physician in a Family Practice training program accidentally injected epinephrine into his left thumb while reading the operating instructions of an autoinjector (Epipen^®^). He developed swelling, pallor, and pain in the thumb. Treatment included topical nitroglycerin, oral vasodilators and warming of the thumb. As expected, none caused an immediate response; however, after 8 hours, the thumb was pink and warm. There was full recovery 2 months after the accident. We reviewed the treatment of accidental epinephrine injection, and found that the use of parenteral adrenergic alpha blocker phentolamine would have produced immediate recovery.

**Conclusions:**

All health professionals concerned with the use of epinephrine autoinjectors should receive adequate instruction on their use. A regimen for management of accidental epinephrine injection, in particular the use of phentolamine, should be emphasized.

## Introduction

Treatment of severe anaphylaxis with epinephrine autoinjector devices such as Epipen^®^ (or Anapen^®^) and Epipen Jr^®^ has been available since 1980. Patients who have an acute allergic reaction can immediately inject epinephrine with them, reversing peripheral vasodilation, edema, constriction of the airways, and myocardial depression. However, these devices are not without risk: myocardial infarction from injected epinephrine has been reported [1]. We report a physician who accidentally injected epinephrine into his thumb while handling an autoinjector, and the results of reviewing the literature.

## Case presentation

**Figure 1:**
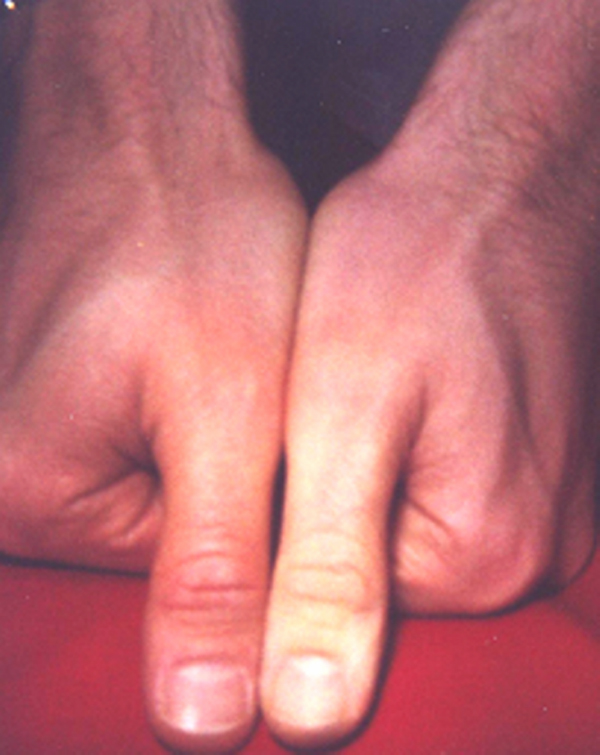
**Delayed capillary refill of the left thumb after accidental injection of epinephrine**.

A 31-year-old physician in a Family Practice training program at an academic primary care center was handling an Epipen^®^ because he wanted to be familiar with it before prescribing it. While reading the operating instructions, he attempted to launch the needle but erroneously placed his left thumb over the needle opening. He accidentally discharged the contents of the spring-loaded syringe into his thumb; and the needle was twisted into the distal tip of his finger. Hand X-ray did not show a fracture of the distal phalanx. He complained of pain and paresthesia in the thumb. The entire digit was cool and pale, and a prolonged capillary refill time was seen at more than 10 s (Figure 1). His blood pressure was 175/85 mmHg, heart rate 80/minute, and respiratory rate 16/minute.

Vascular surgeons, anesthetists, and a hand specialist were contacted by the treating general internist. Several approaches were proposed: metacarpal nerve block or digital block with local lidocaine to induce an inhibition of sympathetic afferents to the digit; and topical nitroglycerin, systemic vasodilators, and warming by immersion in water to increase the blood flow supplying the digit. Topical nitroglycerin, oral vasodilators, and immersion in warm water were attempted unsuccessfully. The intravenous alpha-blocker phentolamine was not prescribed. After 8 hours, the entire finger was pink and warm, either because of the decreasing effect of epinephrine after 90 minutes [2], or because of the delayed effect of the attempted treatment. Full recovery was noted at a 2-month follow-up exam.

## Discussion

The rate of accidental injection of epinephrine from autoinjectors has increased worldwide. The incidence is about 1 per 50,000 Epipen^®^ units [3]. Up to 16% of tested doctors who read the instructions on the Epipen^®^ device self-injected the Epipen^®^ trainer into their thumb. Although no case of digital loss because of an accidental injection of epinephrine has been reported [4], the risk of severe pain and even necrosis due to severe local vasoconstriction is real, so developing formal treatment [5] guidelines is paramount. Immersion in warm water, application of topical nitroglycerin, and nerve blockade have been attempted without success, although Kaspersen *et al.* report some recovery [6]. Subcutaneous injection of terbutaline to reverse vasospasm has been reported once [7].

Since epinephrine induces a vasoconstriction through an alpha adrenergic effect, the use of phentolamine seems logical. Phentolamine is used to control blood pressure during surgery for pheochromocytoma and paraganglioma, when the tumor releases catecholamines because of manipulation and the stress of surgery. Its use is limited by adverse side effects such as hypotension and tachycardia, requiring adequate pre-hydration. Zucker first described phentolamine as useful to prevent necrosis due to levarterenol [8] and Jordan first showed the effectiveness of this drug for accidental epinephrine injection [9]. Others have used local infiltration of accidental epinephrine injection sites successfully [10]-[14].

We think a regimen of treatment should be available to treat accidental epinephrine injection. Velissariou *et al.*[14] successfully used 1.5 mg of phentolamine (from a 10 mg/ml phentolamine mesylate ampoule) diluted in 1 ml of 2% lidocaine to treat such accidents. Velissariou *et al.* injected "*[this mixture] subcutaneously into the site [of accidental epinephrine injection,] and stop as soon as the skin becomes pink*". Peripheral perfusion is restored usually in less than 5 minutes.

Formal training should be provided to all health professionals who prescribe or issue epinephrine autoinjectors. Implicit in epinephrine autoinjector prescriptions is that physicians and pharmacists are confident that the patient or a relative can adequately use the device, and that the patient is aware of the potential dangers of incorrect administration. Thus, those receiving prescriptions for epinephrine autoinjectors should receive similar training. A regimen of management of accidental epinephrine injection emphasizing the use of phentolamine should be instigated.

This event also highlights the importance for all doctors to gain quick access to information in the case of unusual presentation. Access to medical databases through new technologies is essential.

## Consent

Written informed consent was obtained from the patient for publication of this case report and any accompanying images, but is obvious as the patient is the first author of this case report. A copy of the written consent is available for review by the Editor-in-Chief of this journal.

## Competing interests

The authors declare that they have no competing interests.

## Authors' contributions

CM, PS and BF have made substantial contributions to conception, design, writing the manuscript and literature review. All authors read and approved the final manuscript.
